# The Development and Validation of a Glaucoma Health Score for Glaucoma Screening Based on Clinical Parameters and Optical Coherence Tomography Metrics

**DOI:** 10.3390/jcm13226728

**Published:** 2024-11-08

**Authors:** Michael Chaglasian, Takashi Nishida, Sasan Moghimi, Ashley Speilburg, Mary K. Durbin, Huiyuan Hou, Nevin W. El-Nimri, Christopher K. Lee, Anya Guzman, Juan D. Arias, Timothy Bossie, Yu Xuan Yong, Linda M. Zangwill, Robert N. Weinreb

**Affiliations:** 1 Illinois College of Optometry, Chicago, IL 60616, USA; mchaglas@ico.edu (M.C.); ascheurer@ico.edu (A.S.); 2 Hamilton Glaucoma Center, Shiley Eye Institute, Viterbi Family Department of Ophthalmology, University of California, San Diego, CA 92039, USA; tnishida@health.ucsd.edu (T.N.); samoghimi@health.ucsd.edu (S.M.); x1yong@health.ucsd.edu (Y.X.Y.); lzangwill@health.ucsd.edu (L.M.Z.); rweinreb@ucsd.edu (R.N.W.); 3 Topcon Healthcare, Oakland, NJ 07436, USA; mdurbin@topcon.com (M.K.D.); nel-nimri@topcon.com (N.W.E.-N.); clee@topcon.com (C.K.L.); aguzman@topcon.com (A.G.); jarias@topcon.com (J.D.A.); 4 New England College of Optometry, Boston, MA 02115, USA; bossiet@neco.edu

**Keywords:** optical coherence tomography, glaucoma screening, primary eye care, glaucoma health score

## Abstract

**Background/Objectives:** This study aims to develop and validate a Glaucoma Health Score (GHS) that incorporates multiple individual glaucoma risk factors to enhance glaucoma detection in screening environments. **Methods:** The GHS was developed using a retrospective dataset from two clinical sites, including both eyes of glaucoma patients and controls. The model incorporated age, central corneal thickness, intraocular pressure, pattern standard deviation from a visual field threshold 24-2 test, and two parameters from an optical coherence tomography (OCT) test: the average circumpapillary retinal nerve fiber layer thickness and the minimum thickness of the six sectors of the macular ganglion cell plus the inner plexiform layer. The GHS was then validated in two independent datasets: one from primary care sites using Maestro OCT data (test dataset 1) and another from an academic center using DRI OCT Triton (test dataset 2). **Results:** Both eyes of 51 glaucoma patients and 67 controls were included in the development dataset. Setting the GHS cutoff at 75 points out of 100, test dataset 1, which comprised 41 subjects with glaucoma and 41 healthy controls, achieved an area under the receiver operating characteristic curve (AUROC) of 0.98, with a sensitivity of 71% and specificity of 98%; test dataset 2, which included 53 patients with glaucoma and 53 healthy controls, resulted in an AUROC of 0.95, with a sensitivity of 75% and specificity of 96%. A decision curve analysis across all datasets demonstrated a higher net benefit for the GHS model compared to individual OCT parameters. **Conclusions:** The GHS offers a feasible, standardized approach for early detection of glaucoma, providing strong specificity and acceptable sensitivity, with clear decision-making benefits in screening settings.

## 1. Introduction

Glaucoma is one of the leading causes of irreversible blindness worldwide [1]. Open-angle glaucoma, the most common form, is characterized by progressive damage to the optic nerve and gradual vision loss. It typically has no symptoms in its early stages, making it crucial to detect and manage before significant vision impairment occurs. The disease is associated with risk factors such as elevated intraocular pressure (IOP), age, and family history, and it progresses gradually over time if left untreated [2]. The severity of the disease at the time of diagnosis is strongly correlated with outcomes, indicating that earlier detection is imperative [3]. With numerous available glaucoma treatment options, such as pharmacological, laser, and surgical [2], ensuring that patients are routinely screened in primary eye care settings and triaged appropriately for treatment and management is important [3].

While the literature suggests that population screening can identify undetected glaucoma and may reduce low vision and blindness caused by the disease by approximately 50% [3], the evidence supporting widespread screening remains inconclusive [4]. Primary eye care may serve as an effective means for glaucoma detection, as patients come in for routine vision-related services or annual eye examinations. Paul et al. found that integrating optical coherence tomography (OCT) as part of routine eye examinations in primary eye care increased referrals to specialists for glaucoma management [5]. However, they and others have noted that this approach lacks the required specificity, hence creating a scenario where glaucoma specialists are inundated with patients who may not require their care [3,5,6]. For this reason, there is a clinical need for systematic, highly specific methods to screen patients and identify those in need of glaucoma monitoring at the earliest stages possible, such as in primary eye care clinics. These methods would enable effective early detection of glaucoma and appropriate patient triage, thereby preventing vision loss. Given that the majority of individuals who are evaluated in these screening settings do not have glaucoma, such methods must prioritize high specificity. One systematic approach to achieve high specificity, without compromising early detection, is to develop a single score that includes OCT and other clinically relevant risk factors to quantify the likelihood of having glaucoma, facilitating appropriate follow-up and triage.

Several scores based solely on OCT have been developed [7,8,9]. While these may prove useful in screening settings, they exclude other standard clinical parameters that practitioners consider when detecting glaucoma. Methods that incorporate additional clinical parameters have been derived from the Ocular Hypertension Treatment Study (OHTS) [10] and the European Glaucoma Prevention and Study (EGPS) [11]. However, these scores were specifically designed to assess the risk of developing glaucoma in individuals with ocular hypertension [12], are not applicable to patients without this condition, and are hence not applicable for the purpose of screening.

In this study, we aim to develop and evaluate a health score for open-angle glaucoma that utilizes data that are readily available in primary eye care settings, which routinely use OCT for screening of disease, and to assess the likelihood of a patient having glaucoma at the time of their visit.

## 2. Materials and Methods

### 2.1. Model Development

To develop the Glaucoma Health Score (GHS), data were retrospectively evaluated from two primary eye care clinics (the Illinois College of Optometry (ICO) and the Topcon Healthcare Innovation Center (THINC)). Both study protocols received Institutional Review Board (IRB) approval and adhered to the principles of the Declaration of Helsinki and the Health Insurance Portability and Accountability Act.

Subjects were included if 12 × 9 mm 3D Wide OCT scans imaged using the Maestro2 (Topcon Corporation, Tokyo, Japan), from both eyes of the participants, were acceptable and if no confounding ocular pathology (such as epiretinal membrane or diabetic retinopathy) was present. To be considered normal for the model development purposes, both eyes had to be free of elevated intraocular pressure (IOP < 22), structural abnormalities, or functional loss that indicate glaucomatous damage based on the clinical records. To be classified in the glaucoma category, at least one eye had to be diagnosed with glaucoma as per the clinical records of the site based on a comprehensive eye exam. There were no specific requirements for the severity of glaucoma. If a subject was suspected of glaucoma in either eye due to ocular hypertension or a suspiciously appearing disk but did not have a definitive diagnosis of glaucoma in either eye, they were treated as a suspect and included in the control group. The clinical diagnosis at the time of the OCT evaluation, based on a comprehensive eye exam, was used at the subject level. Clinical data and OCT measurements from both eyes of each subject were included. The clinical data evaluated comprised age at the time of the OCT scan, central corneal thickness (CCT), and IOP, as well as the pattern standard deviation (PSD) from a 24-2 threshold visual field test (using either the Humphrey Field Analyzer (HFA, ZEISS, Dublin, CA, USA) or TEMPO/imovifa (Crewt Medical Systems Inc., Tokyo, Japan)). The OCT data (Maestro2) included two diagnostically useful parameters for discriminating between normal eyes and eyes with glaucoma: the average circumpapillary retinal nerve fiber layer (cpRNFL) thickness and the minimum thickness of the six sectors of the macular ganglion cell plus the inner plexiform layer (minGCL+) [7,8,9].

### 2.2. Model Testing

#### 2.2.1. Test Set 1 (Maestro2)

Independent data from three sites—ICO, the New England College of Optometry (NECO), and THINC—collected using the Maestro2 device, were used to form the first test dataset. This dataset was evaluated by two independent professional graders using the Columbia University OCT-based method (CU OCT) [13]. The graders reviewed the RNFL and GCL+ probability maps, the RNFL and GCL+ thickness maps, and the cpRNFL B-scan image on the OCT reports. Scans considered acceptable for categorization were labeled as healthy, optic neuropathy consistent with glaucoma, glaucoma suspects, or other pathologies. Scans labeled as suspects or other pathologies were excluded from the analysis. OCT scans were reviewed for acceptability and excluded if the graders identified artifacts such as clipping and/or segmentation errors that were likely to significantly impact the cpRNFL thickness measurements, evidence of significant motion artifacts, blinks, or mispositioning of the optic disk in the GCL+. Scans with a TopQ (image quality) score of less than 25 were also excluded, as recommended by the Maestro2 user manual for this device and scan type.

Cases where the graders disagreed on a diagnosis were resolved through adjudication in a decision meeting. This process involved reassessing the case with additional clinical data, such as IOP, CCT, visual field reports, and OCT reports from the patient record, including previous visits, if available. Data from both eyes were reviewed and categorized into the healthy or glaucoma groups. Subjects in the healthy group were required to have both eyes labeled as healthy. Subjects in the glaucoma group only required one eye to be labeled as glaucomatous. The severity of the glaucoma of each subject was determined by the worse eye. The healthy and glaucoma groups were matched for sex and age.

#### 2.2.2. Test Set 2 (Triton)

The second independent dataset was from the Hamilton Glaucoma Center, Shiley Eye Institute, University of California, San Diego. The subjects were included from the Diagnostic Innovations in Glaucoma Study (DIGS) [14]. All subjects provided written informed consent, the IRB at the University of California San Diego approved all protocols, and all methods adheres to the Declaration of Helsinki. The methods for diagnosing glaucoma and the OCT device used (DRI OCT Triton, Topcon Corporation, Tokyo, Japan) differed from those in the development dataset and test dataset 1 to verify performance in different settings, considering clinical practice applicability.

The inclusion and exclusion criteria for DIGS were described previously [14]. Specifically, all subjects underwent the following examinations: (1) a baseline examination including ultrasound pachymetry and gonioscopy; (2) an annual ophthalmologic examination including best-corrected visual acuity, slit lamp biomicroscopy, dilated fundus examination, and stereoscopic optic disk photography; and (3) a semiannual examination including IOP measurement with Goldmann applanation tonometry, visual field, and OCT in both eyes. The inclusion criteria also consisted of (1) age above 18 years, (2) open angles on gonioscopy, (3) best-corrected visual acuity of 20/40 or better, and (4) refraction within 5.0 diopters spherical and within 3.0 diopters cylinder at baseline. The exclusion criteria were (1) a history of trauma or intraocular surgery (except for uncomplicated cataract surgery or glaucoma surgery); (2) coexisting retinal disease, uveitis, or non-glaucomatous optic neuropathy; (3) other systemic or ocular diseases known to affect visual field performance or reliability; and (4) a diagnosis of Parkinson disease, Alzheimer disease, or dementia or a history of stroke. Eyes with an axial length of 27 mm or greater were also excluded. Reliable visual fields (fixation losses and false negatives ≤ 33% and false positives ≤ 15%) were assessed using the 24-2 HFA SITA-Standard, and OCT data were derived from the Triton OCT 3D wide scans (12 × 9 mm) encompassing the peripapillary and macula regions. OCT scans with an image quality score of ≥40 [15] were included in this dataset.

Eyes characterized as glaucoma suspect were defined as having glaucomatous optic neuropathy or an elevated IOP of 22 mmHg or greater without any repeatable glaucomatous visual field defect. Eyes with primary open-angle glaucoma were defined by glaucomatous optic neuropathy and repeatable glaucomatous visual field defects, which included a Glaucoma Hemifield Test outside the normal limits and a PSD outside the 95% normal limits [14]. Two masked clinical graders reviewed stereoscopic optic disk photographs for the presence of glaucomatous optic neuropathy based on neuroretinal rim narrowing, notching, excavation, or localized or diffuse RNFL defect [14]. If one eye had glaucoma, the subject was treated as a glaucoma subject, and if both eyes were normal, the subject was treated as normal.

### 2.3. Statistical Analysis

Continuous and categorical data are presented as mean (standard deviation (SD)) and count. The statistical significance of differences across groups was determined by two-sample *t*-tests for continuous variables and chi square tests for categorical variables.

A logistic regression model was fitted to the development data using MATLAB (version: 9.8.0 R2020a, The MathWorks Inc.: Natick, MA, USA) to predict whether the clinical assessment indicated glaucoma as opposed to control. The model was then simplified by combining weights from both eyes to create a model that was applicable to a subject. Since the goal of the tool is to support screening in settings where glaucoma presents at its natural prevalence of 0.6–8.3% [16], a model cutoff that provided a specificity of 95% in the development data was selected. A second cutoff that gave a specificity of 85% in the development data was also considered.

The GHS was applied to the subject of the test dataset, and the area under the receiver operating characteristic curve (AUC) was calculated. To evaluate the sensitivity and specificity, the score was compared to one of two thresholds: one associated with 95% specificity and the other with 85% specificity in the development dataset. The ground truth was determined by the diagnosis generated by the independent graders. Confidence limits at the 95% level were calculated using the exact binomial calculation.

The clinical utility of the GHS, cpRNFL, and minGCL+ were assessed through decision curve analysis (DCA) to determine its net benefit in potential clinical use. Data from the development and test datasets were analyzed using multivariable logistic regression models, normalized across datasets. Binomial generalized linear mixed models were employed with random effects for patients to account for within-subject variability [17].

## 3. Results

The development dataset comprised data from both eyes of 51 subjects with glaucoma and 67 control subjects. Table 1 presents descriptive statistics for the development dataset. Most eyes with glaucoma were in the early stage, with only 16 out of 102 eyes having a mean deviation (MD) worse than −6 decibels (dB). There was no significant difference among the groups in terms of age, IOP, and CCT. The groups differed by gender, MD, PSD, cpRNFL, and minGCL+.

**Figure 1:**
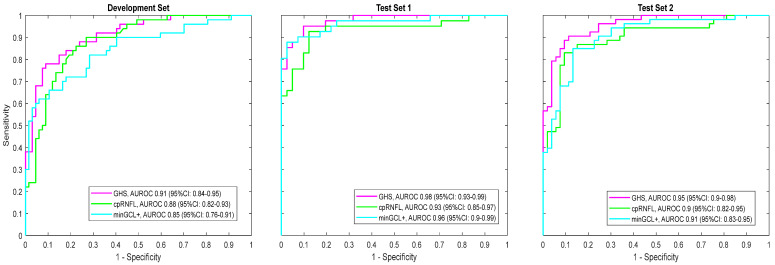
Receiver operating characteristic curves for the development dataset (**left**), test set 1 (**middle**), and test set 2 (**right**) for the Glaucoma Health Score (GHS) (magenta) compared to curves for the average circumpapillary retinal nerve fiber layer (cpRNFL) thickness and minimum thickness of the six sectors of the macular ganglion cell plus the inner plexiform layer (GCL+).

Figure 1 shows the resulting receiver operating characteristic (ROC) curve for the development dataset, test set 1, and test set 2, respectively. They were compared to the ROC curve of two common metrics: the cpRNFL average and the minimum grid value for the ganglion cell plus the inner plexiform layer thickness (minGCL+). In the development dataset, the AUC (with 95% confidence limits) for the GHS was 0.91 (0.84, 0.95), compared to 0.88 (0.82, 0.93) for cpRNFL and 0.85 (0.76, 0.91) for minGCL+.

The logistic regression in Formula (1), based on the model fit and simplified to apply to a single eye (by summing the weights for both eyes), was as follows:

(1) $$Y=11.43-0.017\times \mathrm{Age}+0.138\times \mathrm{IOP}-0.004\times \mathrm{CCT}+0.329\times \mathrm{PSD}-0.083\times \mathrm{cpRNFL}-0.076\times \mathrm{minGCL}+,\;P=\frac{e^{Y}}{1+e^{Y}}\times 100$$

Based on the range of values observed in the development dataset, the factors were ranked in terms of their potential contribution from highest to lowest as follows: average cpRNFL thickness, PSD, IOP, minimum ganglion cell layer (minGCL+) thickness, age, and CCT.

A cutoff of 75 (of 100 possible) was selected to identify glaucoma, based on a goal of 95% specificity in the development dataset. A cutoff of 50 was established for borderline cases to identify an increasing probability of glaucoma, which was consistent with a specificity better than 85% in the development dataset.

Test dataset 1 included 41 subjects with glaucoma (30 early, 8 moderate, and 3 severe glaucoma), and 41 eyes from 41 healthy controls. Table 1 presents the descriptive statistics for the test dataset. The ROC curve for this dataset, compared with the curves for cpRNFL average and the minGCL+, is shown in Figure 1 (middle). The AUC for the GHS was 0.98 (CI: 0.93, 0.99), which was slightly better than the AUC in this dataset for the minGCL+ 0.96 (CI: 0.90, 0.99) and cpRNFL 0.93 (CI: 0.85, 0.97). Using a cutoff of 75, the sensitivity in the test dataset was 71%, with a specificity of 98%. Using a lower cutoff of 50 to indicate potentially borderline cases, the sensitivity changed to 83%, with the specificity remaining 98%.

In test dataset 2, comprising 53 patients with glaucoma and 53 healthy controls, setting the GHS cutoff at 50 points out of 100 yielded a sensitivity of 89% and a specificity of 91%. Adjusting the GHS cutoff to 75 points resulted in a sensitivity of 75% and a specificity of 96%. The AUC for GHS was 0.95 (95% CI: 0.90 to 0.98). The ROC curve for dataset 2, compared with the curves for cpRNFL average and the minGCL+, is shown in Figure 1 (right).

**Figure 2:**
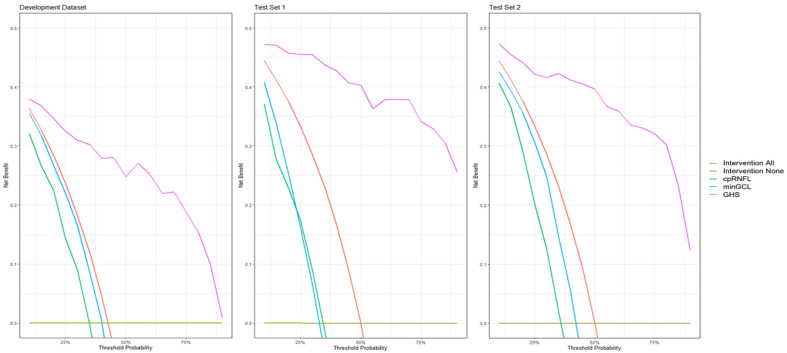
Decision curve analysis for development set (**left**), test set 1 (**middle**), and test set 2 (**right**), showing the baselines “intervene for all” in red and “intervene for none” in olive drab, as well as the curves of cpRNFL in cyan, minGCL+ in green, and GHS in magenta.

Figure 2 shows the decision curves for all three datasets, comparing GHS, cpRNFL and minGCL+ to “intervene for none” and “intervene for all” scenarios. The DCA results demonstrate that the GHS consistently provides the highest net benefit compared to cpRNFL and minGCL+ across all decision thresholds. Specifically, the GHS curve remains above both the “intervene for none” and “intervene for all” reference lines across all thresholds, indicating its superior clinical utility.

## 4. Discussion

This study presents a model, the Glaucoma Health Score (GHS), which uses OCT and clinical parameters to predict the likelihood of glaucoma in screening settings.

The GHS uses a logistic regression model that is similar to the OHTS and EGPS scores [11,12] but includes subjects with a wider range of IOP. Additionally, it incorporates OCT analysis/data, which were not available at the time of the initial OHTS and EGPS studies.

The GHS is similar to several OCT-only models, such as the UNC OCT Index [9] by Fukai et al. [7], in that it outperforms single metrics derived from OCT data. These models were validated in independent datasets and showed strong performance in diagnosing glaucoma, with the UNC OCT Index achieving an AUC of 0.96 and Fukai-Nakano’s models achieving an AUC of 0.97. The GHS has an AUC of 0.98 in the test dataset, which is comparable to these OCT-only models. However, since glaucoma detection is known to be influenced by population characteristics such as age, race, ethnicity, and the severity of glaucoma [18], comparing AUC values across studies or datasets that involve different subject groups may lead to inaccurate conclusions. Therefore, direct comparisons should be avoided unless the subjects in the studies or datasets are closely matched. Overall, the GHS performs similarly to these OCT-only models. However, the GHS improves upon these earlier models by incorporating clinical parameters that are commonly used in the diagnosis of glaucoma, as it would be advantageous for clinicians in screening settings to consider more than just OCT [11].

The GHS benefits from incorporating clinical parameters that practitioners collect in screening settings and consider when making decisions. These parameters are also known to be associated with the onset and progression of glaucoma [2,10,11]. Previously developed glaucoma risk scores using clinical data generally did not include OCT or included OCT only and were designed specifically for clinically indicated populations rather than for use in screening settings. These risk scores are intended to support decisions regarding glaucoma treatment in the presence of a high IOP [10,11]. Consequently, their performance is not measured based on a binary comparison and their performance cannot be directly compared to the GHS. Conversely, the clinical decision supported by the GHS is whether or not a patient should be monitored for glaucoma. While the incidence of undiagnosed glaucoma remains high, the prevalence of glaucoma in patients seeking vision-related care or annual eye examinations has been demonstrated to be low, so it is essential to optimize specificity to avoid false positives overwhelming the healthcare system [19]. The high specificity observed in the test dataset suggests that the GHS could be useful in these scenarios. The introduction of a high-specificity tool to screen for the possible presence of glaucoma could help prevent false positives that have been observed using OCT in these clinical settings [5,19,20,21]. The superior clinical utility of the GHS is also demonstrated by the high net benefit shown for the GHS in the DCA [22]. This suggests that incorporating the GHS into screening protocols could improve the accuracy of glaucoma detection, potentially reducing both false positives and unnecessary interventions. By providing a clearer benefit in decision-making compared to traditional measures, the GHS may help streamline early detection efforts, particularly in screening scenarios, leading to more timely and targeted interventions for individuals at risk. Although the DCA shows that cpRNFL and minGCL+ perform worse than an “intervene for all” strategy, they may still hold potential value in targeted screening strategies for high-risk populations, such as those with high polygenic risk scores, older individuals, or individuals of African or Hispanic descent. In cases where demographic or ophthalmic data (such as IOP, CCT, and VF) included in the GHS are not easily obtainable, future research is needed to assess whether OCT-only parameters, like cpRNFL and minGCL+, could help reduce costs while maintaining screening effectiveness in targeted settings [23,24]. A notable finding of the current study is the effective performance of a score developed in SD-OCT when applied to SS-OCT data. This success can be anticipated due to recent studies showing excellent agreement between measurements from these two devices [25].

Incorporating suspects within the control group of the development set was aimed at capturing the full spectrum of clinical presentations that are encountered in primary eye care settings. By including these suspicious cases within the control group, we aimed to improve the model’s ability to differentiate potential normal variations from those with early signs of glaucoma. This approach helped ensure that the model could handle the inherent variability in real-world patient populations. Furthermore, the GHS demonstrated robust performance across both test datasets, despite variations in data sources, including practice type, patient population, devices used, diagnostic criteria, etc. These findings suggest that the GHS can detect glaucoma effectively across diverse clinical settings, highlighting its broad applicability in real-world scenarios.

One limitation of this study is the small sample size used for development and inability to check the generalizability across age, race, and glaucoma severity. However, a strength of this study is the use of two independent test datasets obtained from two different settings to validate the performance of the model.

Another limitation of this study is the incorporation of suspects within the control arm of the development set, along with the exclusion of suspects from both test sets, which may have contributed to the higher specificity observed in the test sets compared to the development set. Nevertheless, given the necessity for high specificity and the ability of OCT to capture disease earlier than traditional eye examinations that do not use OCT, it seems reasonable to acknowledge that detecting suspects falls beyond the scope of screening.

A third limitation is the use of only global parameters from OCT. Future modifications may include adding local OCT parameters, such as the quadrant RNFL and GCL+ thickness values. However, the introduction of these additional local variables reduces the specificity. A final limitation is the use of parameters such as the CCT and visual field threshold tests that may not be routinely captured in these screening environments. Future work can explore the use of reduced parameter sets.

## 5. Conclusions

Overall, this study demonstrates that it is feasible to develop a multi-risk factor Glaucoma Health Score that includes OCT and maintains excellent specificity and acceptable sensitivity in screening settings. Moreover, the DCA demonstrates that the GHS may be useful in screening settings that include OCT in pre-test examinations, enabling the clinician to determine if further evaluation for glaucoma is indicated.

## Figures and Tables

**Table 1:** Descriptive statistics [mean (standard deviation)] for each parameter considered as part of the screening score (development dataset), test set 1, and test set 2.

	Development Dataset			Test Dataset 1 (Maestro2)			Test Dataset 2 (Triton)		
	Control	Glaucoma	*p*-Value	Control	Glaucoma	*p*-Value	Control	Glaucoma	*p*-Value
Subject (n)	67	51		41	41		53	53	
Age (years)	61.0 (12.6)	60.4 (11.7)	0.8	63.3 (14.2)	63.7 (14.9)	0.92	67.5 (1.9)	70.5 (1.7)	0.233
Male: Female	22:45	27:24	0.03	16:25	16:25	0.99	15:38	15:38	1.000
logMAR	0.0 (0.1)	0.0 (0.1)	0.02	0.0 (2.6)	−0.1 (6.7)	0.01	0.0 (0.1)	0.0 (0.1)	0.038
IOP (mmHg)	17.2 (4.8)	21.9 (6.4)	<0.001	14.6 (2.9)	15.9 (4.5)	0.001	13.6 (2.5)	14.0 (4.2)	0.560
CCT (µm)	547.8 (42.4)	541.6 (33.4)	0.38	543.4 (34.9)	524.4 (36.0)	0.02	544.4 (38.7)	538.3 (40.9)	0.438
MD (dB)	−0.8 (1.9)	−3.5 (4.8)	0.001	0.0 (1.7)	−4.6 (5.0)	<0.001	−0.1 (1.1)	−5.6 (6.0)	<0.001
PSD (dB)	1.8 (1.3)	3.5 (3.2)	<0.001	1.6 (1.4)	4.4 (4.0)	<0.001	1.7 (0.3)	5.7 (3.4)	<0.001
cpRNFL (µm)	99.5 (13.4)	79.4 (17.4)	<0.001	102.7 (10.6)	74.2 (16.8)	<0.001	99.9 (12.2)	72.2 (18.5)	<0.001
minGCL+ (µm)	63.6 (6.9)	52.3 (11.7)	<0.001	65.9 (5.3)	48.0 (7.8)	<0.001	64.0 (6.1)	52.0 (8.8)	<0.001
Severity of Glaucoma									
Early MD ≥ −6 dB Moderate −12 ≤ MD < −6 dB Severe MD ≥ −12 dB	N/A	39 (76%) 5 (10%) 7 (14%)		N/A	30 (73%) 8 (20%) 3 (7%)		N/A	30 (57%) 10 (19%) 13 (25%)	

Abbreviations: logMAR = Logarithm of the Minimum Angle of Resolution; IOP = intraocular pressure; CCT = central corneal thickness; MD = mean deviation; PSD = pattern standard deviation; cpRNFL = circumpapillary retinal nerve fiber layer; minGCL+ = minimum thickness of the ganglion cell layer plus inner the plexiform layer.

## Data Availability

Datasets are available on request from the authors.
